# Nordihydroguaiaretic Acid Attenuates the Oxidative Stress-Induced Decrease of CD33 Expression in Human Monocytes

**DOI:** 10.1155/2013/375893

**Published:** 2013-02-24

**Authors:** Silvia Guzmán-Beltrán, José Pedraza-Chaverri, Susana Gonzalez-Reyes, Fernando Hernández-Sánchez, Ulises E. Juarez-Figueroa, Yolanda Gonzalez, Karen Bobadilla, Martha Torres

**Affiliations:** ^1^Departamento de Microbiología en Investigación, Instituto Nacional de Enfermedades Respiratorias, Ismael Cosío Villegas, Calzada de Tlalpan 4502, Sección XVI, 14080 México, DF, Mexico; ^2^Departamento de Biología, Facultad de Química, Universidad Nacional Autónoma de México (UNAM), Ciudad Universitaria, 04510 México, DF, Mexico

## Abstract

Nordihydroguaiaretic acid (NDGA) is a natural lignan with recognized antioxidant and beneficial properties that is isolated from *Larrea tridentata*. In this study, we evaluated the effect of NDGA on the downregulation of oxidant stress-induced CD33 in human monocytes (MNs). Oxidative stress was induced by iodoacetate (IAA) or hydrogen peroxide (H_2_O_2_) and was evaluated using reactive oxygen species (ROS) production, and cell viability. NDGA attenuates toxicity, ROS production and the oxidative stress-induced decrease of CD33 expression secondary to IAA or H_2_O_2_ in human MNs. It was also shown that NDGA (20 **μ**M) attenuates cell death in the THP-1 cell line that is caused by treatment with either IAA or H_2_O_2_. These results suggest that NDGA has a protective effect on CD33 expression, which is associated with its antioxidant activity in human MNs.

## 1. Introduction

Nordihydroguaiaretic acid (NDGA) is a natural lignan that is primarily isolated and commercially produced from the desert creosote bush, *Larrea tridentata*, which has long been used in traditional medicine for the treatment of several illnesses including diabetes and inflammation [1]. It is estimated that NDGA comprises approximately 5% to 10% of the dry weight of the leaves, and this corresponds to 80% of all the phenolic compounds in the resin [2]. Cell culture and animal model studies have demonstrated that NDGA has biological properties, including anticarcinogenic, antidiabetic, antiviral, antioxidant, and anti-inflammatory activities [3].

The beneficial effects of NDGA have been essentially attributed to its antioxidant properties. NDGA is an effective *in vitro* scavenger of peroxynitrite, singlet oxygen, hydroxyl radical (^•^OH), and hypochlorous acid [4, 5]. It has been shown that NDGA is capable of protecting rats that are exposed to oxidative stress induced by ozone, potassium dichromate, and cisplatin [4, 6, 7]. In addition NDGA also protects primary rat neuronal cultures against damage that is generated by hydrogen peroxide (H_2_O_2_) and iodoacetate (IAA) [8–10]. 

Additionally, it has been found that NDGA induces transcription factor Nrf2 and expression of heme oxygenase-1 (HO-1) in different kinds of line cells [9, 11]. In fact, Nrf2 factor controls the expression of more than 100 genes of cytoprotective proteins including antioxidant enzymes such as HO-1 [11].

On the other hand, it is well established that oxidative stress is implicated in pathologies such as cancer, diabetes, and inflammation. Oxidative stress is an imbalance in the redox state that is generated by exacerbated ROS production or diminished protective systems, such as enzymes or scavenger molecules [12]. In fact, the increased production of reactive oxygen species (ROS) causes cell damage and even cell death, and antioxidants may help to prevent or alleviate diseases in which oxidative stress is involved.

Glutathione is the most abundant nonprotein sulfhydryl compound and the major intracellular redox buffer in almost all cells. This molecule constitutes the first line of the cellular defense mechanism against oxidative injury [13]. There are evidences that the intracellular redox status regulates various aspects of cellular function and that glutathione (GSH) is important in immune modulation [14, 15]. Recently, it has been described in mice that the pretreatment of NDGA before the treatment with the tumor promoting agent 12-O-tetradecanoylphorbol-13-acetate (TPA) mitigated cutaneous lipid peroxidation and inhibited H_2_O_2_ production. NDGA also was able to restore GSH level and activities of antioxidant enzymes and even to attenuate inflammation [16].

H_2_O_2_ and IAA are toxic compounds that are utilized commonly to induce oxidative stress in cell models [8–10]. IAA is an alkylating agent that irreversibly inhibits the glycolytic enzyme glyceraldehyde-3-phosphate dehydrogenase (GAPDH) [17]. IAA reduces adenosine triphosphate (ATP) levels and cell survival in a dose-dependent manner [18]. It has been shown that IAA-induced toxicity is related to ROS production, at least in the hippocampal and cerebellar granule neurons of rats [19].

As an ROS, H_2_O_2_ is less reactive; however, it can easily penetrate cell membranes and react with transition metal ions to produce ^•^OH. This intermediary metabolite reacts rapidly and indiscriminately with biomolecules of all classes, including nucleic acids, free nucleotides, proteins, lipids, and carbohydrates. ROS induce oxidative damage, which may cause DNA mutations, protein inactivation, and cell death [12].

In contrast, monocytes (MNs) play a central role in inflammation and host defense against microorganisms. However, in oxidative stress-related diseases, such as diabetes or atherosclerosis, MNs are permanently activated and produce high levels of ROS and the proinflammatory cytokines IL-6, IL-1, and tumor necrosis factor alpha (TNF-*α*). This increased ROS production may lead to severe disorders, such as chronic inflammation and even cell death [20].

In this context, it has been suggested that changing glutathione redox status, which is the balance between intracellular reduced (GSH) and oxidized (GSSG) glutathione, in antigen presenting cells (APCs) regulates the helper T-cell type 1 (Th1)/Th2 balance due to the production of IL-12 in mice [21].

In addition, after an oxidative challenge, macrophages, mesangial cells, and monocytes increase the amount of available arachidonic acid, by means of the activation of the phospholipases A (PLA) and C (PLC) [22–24]. The arachidonic acid is the substrate for the synthesis of eicosanoids: prostaglandins (PG), leukotrienes, and thromboxanes, which are involved in the inflammatory responses through the production of IL-8 and TNF-*α*. The metabolic conversion of arachidonic acid into its byproducts requires the catalytic activity of cyclooxygenase (COX) or lipoxygenase (LOX), and it is known that oxidant molecules can induce the synthesis of the COX through a transcriptional mediated mechanism, involving the I*κ*B*α* degradation and NF*κ*B nuclear translocation. Therefore the oxidative stress has a dual role in the eicosanoid production; at one hand it is the signal to increase the substrate availability, and on the other hand it activates the biosynthetic pathway [25].

Moreover, diabetic patients with hyperglycemia present oxidative stress and constant inflammation. This is due to diverse mechanisms that are associated with excessive ROS production, such as the irreversible production of advanced glycation end products (AGEs). AGEs stimulate the production of inflammatory cytokines in monocytes and macrophages [26]. Additionally, hyperglycemia may stimulate the production of inflammatory cytokines, such as IL-6, IL-1, and tumor necrosis factor alpha (TNF-*α*), by increasing the levels of peroxides and free radicals and inducing inflammation [27]. Recently, the decreased expression of CD33 has been described in the macrophages of diabetic patients with hyperglycemia. This disorder contributes to the spontaneous secretion of TNF-*α*, and this alteration may promote additional inflammation during the early stages of diabetes mellitus type II [28]. 

CD33 is a myeloid cell-specific type I transmembrane glycoprotein that is constitutively expressed on the surfaces of both myeloid progenitors and mature monocytes. This molecule is a receptor that belongs to the family of sialic acid-binding immunoglobulin Ig-like lectins (SIGLECS) [29].

In this study, we evaluate the potential protective effect of NDGA on IAA- and H_2_O_2_-induced toxicity in the THP-1 cell line and in human MNs. We also demonstrate that NDGA attenuates the oxidant stress-induced CD33 expression downregulation in human MNs.

## 2. Materials and Methods 

### 2.1. MNs and THP-1 Cells

Peripheral blood mononuclear cells were obtained from heparinized venous whole blood by gradient centrifugation over a Ficoll-sodium diatrizoate solution (Lymphoprep, Nycomed Pharma, Oslo, Norway) using standard procedures [30]. The layer containing the peripheral blood mononuclear cells (PBMCs) was harvested, and the MNs were enriched by plastic adherence for 1 h at 37°C. The human peripheral blood samples were obtained from the blood bank at the Instituto Nacional de Enfermedades Respiratorias (INER) under approbation of the Institutional Ethical Review Board of INER. 

The human MNs were cultured in RPMI 1640 medium (Cambrex, Walkersville, MD, USA) supplemented with 50 *μ*g/mL gentamicin sulfate, 2.0 mmol/L L-glutamine, and 10% heat-inactivated pooled human serum at 37°C in a 5% CO_2_ atmosphere (MN medium). 

The human acute monocytic leukemia cell line, THP-1, was purchased from the American Type Culture Collection (TIB202, ATCC, Rockville, MD, USA). THP-1 cells were grown in RPMI 1640 supplemented with 10% fetal bovine serum (Lonza, Walkersville, MD, USA), 0.05 *μ*M *β*-mercaptoethanol, 4 mM L-glutamine, 100 U/mL penicillin, and 100 *μ*g/mL streptomycin (THP medium).

### 2.2. Effect of NDGA on Cell Viability

The MNs and THP-1 cells were seeded at 3 × 10^5^ cells/well in 48-well plates in culture medium. The NDGA (5–50 *μ*M) was then added to the cells and incubated for 120 h. After incubation, the cell viability was quantified by MTT reduction. 

### 2.3. Effect of Iodoacetate or H_2_O_2_ on Cell Viability

The human MNs and THP-1 cells (3 × 10^5^ cells/well) were cultured in 48-well plates. First, the cells were treated with 0–100 *μ*M IAA or 0–20 mM H_2_O_2_ for 2 h to induce oxidative stress. Successively oxidants were replaced by fresh medium, and the incubation was continued until 24 h. Cell viability was monitored by MTT reduction or trypan blue exclusion. In addition cell morphology was observed in bright field micrographs on 40x with phase contrast microscopy (Nikon Co.).

### 2.4. Protective Effect of NDGA on the Cytotoxicity-Induced by IAA or H_2_O_2_ in MN and THP-1 on Cell Viability

MNs and THP-1 cells (3 × 10^5^ cells/well) were cultured in 48-well plates (MN or THP medium, resp.). The cells were pretreated with or without 20 *μ*M NDGA by 12 h, and then cells were exposed to (0–100 *μ*M) IAA or (0–20 mM) H_2_O_2_ for 2 h to induce oxidative stress and refreshed after removal of the toxic compounds. MTT reduction or trypan blue exclusion was determined 24 h after onset of IAA or H_2_O_2_ exposure.

### 2.5. Cell Viability Detection by MTT

Cell viability was determined using the 3-(4,5-dimethylthiazol-2-yl)-2,5-diphenyltetrazolium bromide (MTT; Sigma, St. Louis, MO) reduction assay [31]. Briefly, MNs (3 × 10^5^ cells/well) were seeded into 48-well plates; after the indicated treatments and time periods, 50 *μ*L MTT stock solution (5.0 mg/mL) was added to the cells and incubated for 2 h at 37°C in a 5% CO_2_ atmosphere. The plates were then centrifuged at 1,500 g for 15 min at room temperature (RT), and the medium was carefully removed by aspiration. Subsequently, an acid isopropanol solution (800 *μ*L) was added to the wells, and the plates were shaken at 80 rpm for 5 min. Finally, the absorbance was measured at 570 nm in a microplate reader (Labsystems Multiskan). The number of viable cells was expressed as the index of MTT reduction. The control cells (without treatment) were assigned a maximum value of 1, and the indices of the cells incubated with the different treatments were obtained with respect to the control cells.

### 2.6. Cytotoxicity Assay by Trypan Blue Exclusion

Cell viability also was monitored by trypan blue negative cells. Concisely, an aliquot on the cell suspension was diluted 1 : 1 (v/v) with 0.4% trypan blue, and the cells were counted with a hemocytometer [32]. The results represent the percentage with respect to control of trypan blue negative cells (without treatment). This assay was done three times for independent experiments. 

### 2.7. Determination of Reactive Oxygen Species (ROS) Production by Flow Cytometry

#### 2.7.1. IAA Treatment

 MNs (5 × 10^5^ cells/well) were cultured in 24-well plates. First, the cells were pretreated with or without 20 *μ*M NDGA for 12 h and immediately exposed to nonlethal concentration of IAA (2.5, 5.0 and 7.5 *μ*M) by 48 h. Then, ROS production was determined.

#### 2.7.2. H_2_O_2_ Treatment

 MNs (5 × 10^5^cells/well) were pretreated with or without 20 *μ*M NDGA for 12 h and then exposed to H_2_O_2_ (0.5, 1.0 and 2.0 mM) by 12 h. Then, ROS production was determined.

ROS detection was measured with the fluorescent marker 5-(and-6)-carboxy-2′,7′-dichlorodihydrofluorescein diacetate (carboxy-DCFDA; Invitrogen, Carlsbad, CA), which is an acetylated form of fluorescein, and was used as an ROS indicator [33]. 

After treatments, the MNs were washed twice with phosphate-buffered saline solution (PBS) and immediately loaded with 10 *μ*M carboxy-H_2_DCFDA for 30 min at 37°C in the dark for ROS production. Following this incubation, the cells were centrifuged at 600 g for 5 min. The cells were washed twice with PBS and then fixed with 1% paraformaldehyde and stored at 4°C until acquisition with a FACSCalibur flow cytometer (BD, San Jose, CA, USA). The number of events acquired was 10,000. 

### 2.8. Determination of CD33 Expression by Flow Cytometry

#### 2.8.1. IAA Treatment

 MNs (5 × 10^5^cells/well) were cultured in 24-well plates. First, the cells were pretreated with or without 20 *μ*M NDGA for 12 h and immediately exposed to no lethal concentration of IAA (2.5, 5.0 and 7.5 *μ*M) by 72 h. Then, CD33 expression was determined.

#### 2.8.2. H_2_O_2_ Treatment

 MNs (5 × 10^5^cells/well) were pretreated with vehicle or 20 *μ*M NDGA for 12 h and then, exposed to H_2_O_2_ (0.5, 1.0 and 2.0 mM) by 24 h. Then, CD33 expression was determined.

To measure CD33 in cell surface of MNs, the cells were washed with PBS and immediately loaded with saturating amounts of phycoerythrin- (PE-) labeled mAbs against CD33 and incubated for 15 min at RT in the dark. The cells were fixed with 1% paraformaldehyde and stored at 4°C until acquisition. The number of events acquired was 10,000.

The results of ROS level and CD33 expression were analyzed with CellQuest software (BD Biosciences) and were expressed as the index of the mean fluorescence intensity (MFI) compared to the control without treatment.

### 2.9. Glutathione/Glutathione Disulfide Ratio Detection

THP-1 cells (3 × 10^6^ cells/well) were cultured in 6-well plates. First, the cells were pretreated with vehicle or 20 *μ*M NDGA for 12 h and immediately exposed to IAA (2.5, 5.0 and 7.5 *μ*M) or H_2_O_2_ (0.5, 1.0 and 2.0 mM) by additional 24 h. Then, glutathione was measured. 

GSH and GSSG levels were measured in cell extracts using the GSH reductase enzyme method [34]. This assay is based on the reaction of GSH with 5, 5′-dithio-bis (2 nitrobenzoic acid) (DNTB) to form 5-thio-2-nitrobenzoic acid (TNB), detectable at *λ* = 412 nm. The test is specific to GSH on the basis of the specificity of the GSH reductase enzyme to GSH: the rate of accumulation of TNB is proportional to the concentration of GSH in the sample. For this assay, the cells extract was diluted 1 : 1 with KPE buffer prior to addition of freshly prepared DTNB and GSH reductase solutions. Following addition of *β*-NADPH, the absorbance was measured immediately and at 30 s intervals for 1.5 min. The rate of change in absorbance was compared to that of GSH standards. The measurement of GSSG in each sample was identical to that used for the measurement of GSH, but with a previous treatment of each sample with 2-VP, which reacts out with GSH.

### 2.10. Statistics

The data were analyzed with Prism 5 software (GraphPad, San Diego, CA) using a two-way analysis of variance (ANOVA) followed by the Bonferroni multiple comparison test or with a one-way ANOVA followed by the Dunnett's test, as appropriate. A value of *P* < 0.05 was considered significant. 

## 3. Results

### 3.1. Effect of NDGA, IAA and H_2_O_2_ in Human Monocytes on Cell Viability

**Figure 1:**
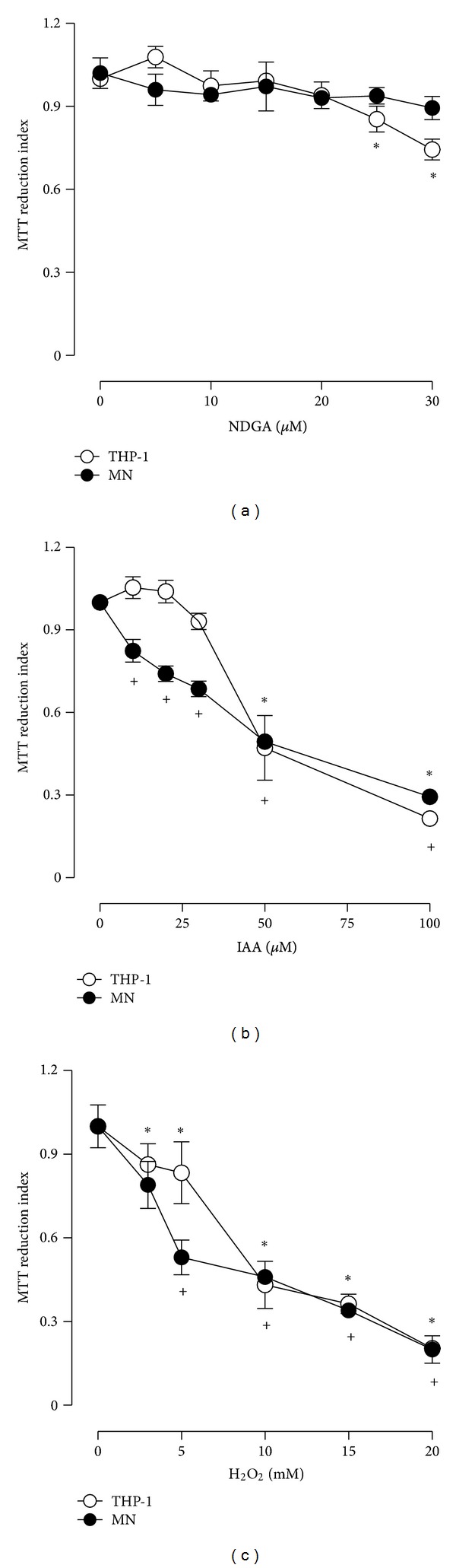
Effect of (a) NDGA, (b) IAA, and (c) H_2_O_2_ on the viability of human MN and THP-1 cells. (a) NDGA (0–30 *μ*M) was added for 120 h before the measurement of viability. In addition, cells were exposed to (b) IAA (0–100 *μ*M) or (c) H_2_O_2_ (0–20 mM) for 2 h. At the end of the exposition time, the media containing IAA and H_2_O_2_ were replaced with fresh medium. Viability was assessed at the end of 24 h of incubation. The number of viable cells is expressed as index of MTT reduction. Data are expressed as mean ± SEM; *n* = 5.  *P < 0.05 versus MN (without treatment) and  ^+^ *P* < 0.05 versus THP-1 (without treatment). MN: human monocytes; THP1: human acute monocytic leukemia cell line.

First, we evaluated the effect of NDGA on cell viability and determined that NDGA did not decrease the cell viability of MNs at concentrations ranging from 5 to 30 *μ*M (Figure 1(a)); however, the viability of THP-1 cells was slightly decreased at NDGA concentrations of 25 and 30 *μ*M after 120 h incubation. Therefore, the concentration of 20 *μ*M was chosen to evaluate the potential antioxidant effect of NDGA against the damage induced by IAA and H_2_O_2_ on MNs and THP-1 cells. 

We also evaluated the effects of different concentrations of IAA and H_2_O_2_ on cell viability. The cells were incubated with these oxidants just by 2 h; then the compounds were removed and fresh medium with 20 *μ*M NDGA was added. The cells were incubated until 24 h and viability quantified. As expected, IAA caused a dose-dependent decrease in the viability of the MNs and THP-1 cells. The MN viability decreased progressively and was significant at a concentration of 10 *μ*M IAA. The THP-1 cell viability decreased significantly at 50 to 100 *μ*M (Figure 1(b)). At the same way, H_2_O_2_ (2.5–20 mM) caused cell death in a concentration-dependent manner. MN cell death increased progressively from 5.0 to 20 mM H_2_O_2_. THP-1 cell death was substantial and increased significantly from 2.5 to 20 mM H_2_O_2_ (Figure 1(c)). 

We also verified THP-1 cell morphology in bright field micrographs. These cells were round and bright throughout the field when treated with vehicle or 20 *μ*M NDGA (Figure 3). Nevertheless, IAA (25–100 *μ*M) and H_2_O_2_ (5–20 mM) induced morphological alterations, such as loss of shape round. Both oxidants were able to generate damage on the cells which were dependent on concentrations (Figures 3(a) and 3(b) left side).

**Figure 2:**
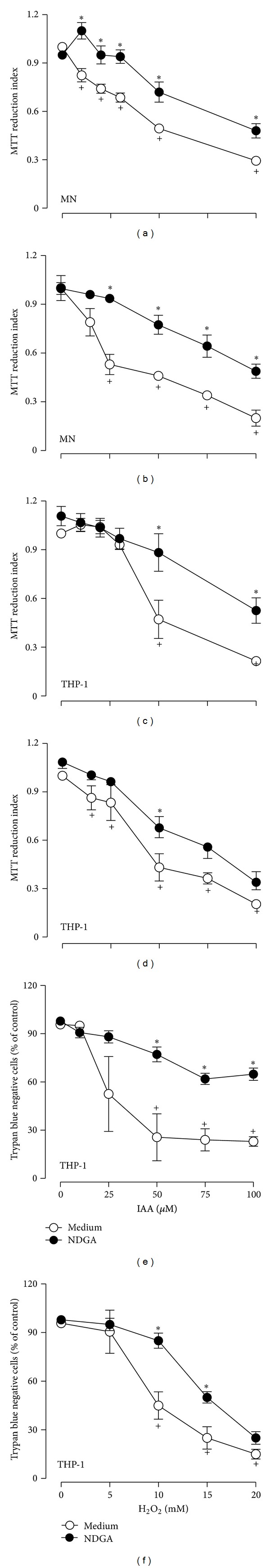
Protective effect of NDGA on the cytotoxicity-induced by ((a), (c), and (e)) IAA and ((b), (d), and (f)) H_2_O_2_ in human cells. ((a), (b)) MN and ((c), (d), (e), (f)) THP-1 cells were pretreated in absence or presence of 20 *μ*M NDGA by 12 h. Cell cultures were exposed to IAA or H_2_O_2_ for 2 h and refreshed after removal of the toxic compounds. MTT reduction or trypan blue exclusion was determined 24 h after the onset of IAA or H_2_O_2_ exposure. The number of viable cells is expressed as ((a), (b), (c), (d)) index of MTT reduction or ((e), (f)) percentage of trypan blue negative cells. Data are expressed as mean ± SEM; *n* = 5.  ^+^ *P* < 0.05 versus control (without IAA or H_2_O_2_) and  *P < 0.05 versus control (without NDGA).

We then assessed the protective effect of NDGA on MNs and THP-1 cells cultured in the presence of IAA and H_2_O_2_ (Figure 2). Our results demonstrate that cell death was decreased in the MNs at all concentrations of IAA (Figure 2(a)), and the NDGA avoided the death of the THP-1 cells by 31% and 41% at 50 *μ*M and 100 *μ*M IAA, respectively (Figure 2(c)). In addition, pretreatment with NDGA decreased cell damage by H_2_O_2_, and cell death was significantly diminished over a range of 5–20 mM H_2_O_2_ (Figure 2(b)). For the THP-1 cells, the protective effect of NDGA was significant at 10 mM H_2_O_2_ (Figure 2(d)). These results also were comparable with percentages of trypan blue negative cells (Figures 2(e) and 2(f)).

**Figure 3:**
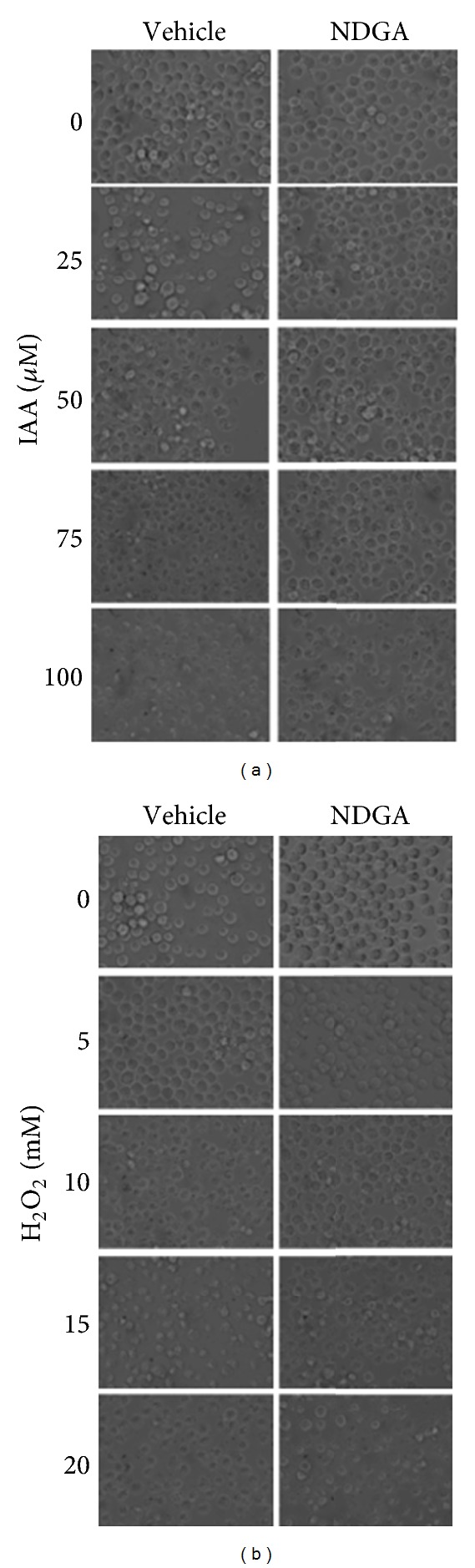
Representative phase contrast micrographs showing the effect of NDGA on the damage induced by different concentrations of (a) IAA or (b) H_2_O_2_ in THP-1 cells. Cells were treated with vehicle (left side) or 20 *μ*M NDGA (right side) by 12 h; after this the cells were exposed to IAA or H_2_O_2_ for 2 h. After this time these compounds were withdrawn and fresh medium with NDGA was added. Representative images were obtained 24 h after the onset of IAA or H_2_O_2_ exposure.

Likewise these results were corroborated observing cell morphology in bright field micrographs (Figure 3). Cells incubated with 20 *μ*M NDGA diminished significantly damage in cell morphology when cells were treated with IAA (25 and 50 *μ*M) or H_2_O_2_ (5 and 10 mM). But the cells pretreated with NDGA and highest concentration of IAA (100 *μ*M) or H_2_O_2_ (15 and 20 mM) still showed cell damage (Figures 3(a) and 3(b) and right side).

### 3.2. Determination of ROS Production Induced by IAA and H_2_O_2_ in Human Monocytes

**Figure 4:**
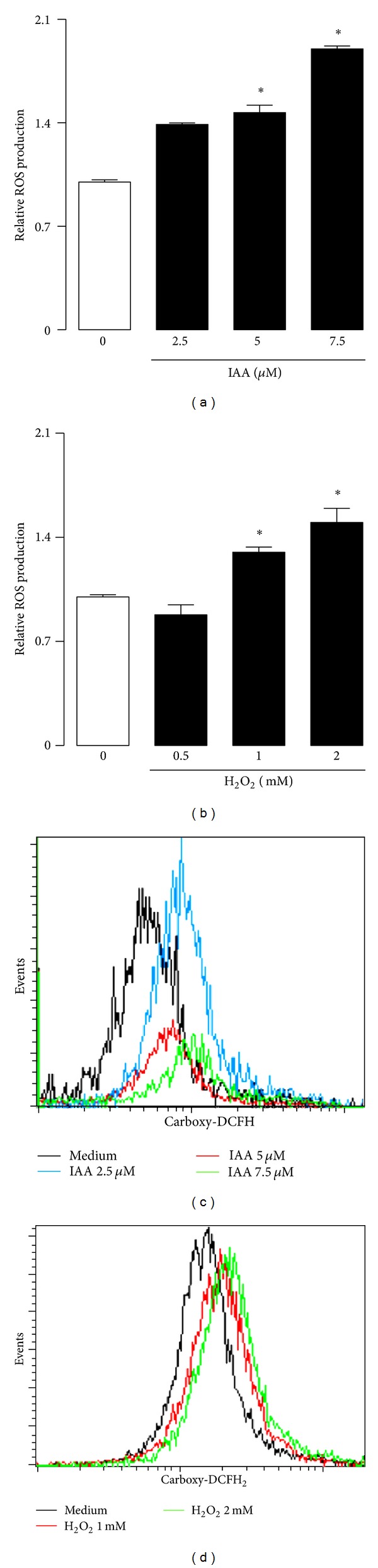
Effect of ((a), (c)) IAA or ((b), (d)) H_2_O_2_ on ROS generation in MN. Cells were treated 48 h with IAA by 12 with H_2_O_2_. Then, ROS production was determined by flow cytometry. ROS level was expressed as index with respect to control without treatment. Data are expressed as mean ± SEM; *n* = 5.  *P < 0.05 versus control (without IAA or H_2_O_2_).

In this study, we showed that low IAA concentration caused a significant increase of ROS during at 48 h incubation (Figures 4(a) and 4(c)). The relative ROS production was increased for all of the IAA concentrations tested (2.5 to 7.5 *μ*M). Similarly, H_2_O_2_ induced a significant increase in ROS production (Figures 4(b) and 4(d)) at concentrations of 1.0 and 2.0 mM after at 12 h incubation.

### 3.3. Glutathione Level in THP-1 Cells Induced by NDGA, IAA, and H_2_O_2_

**Figure 5:**
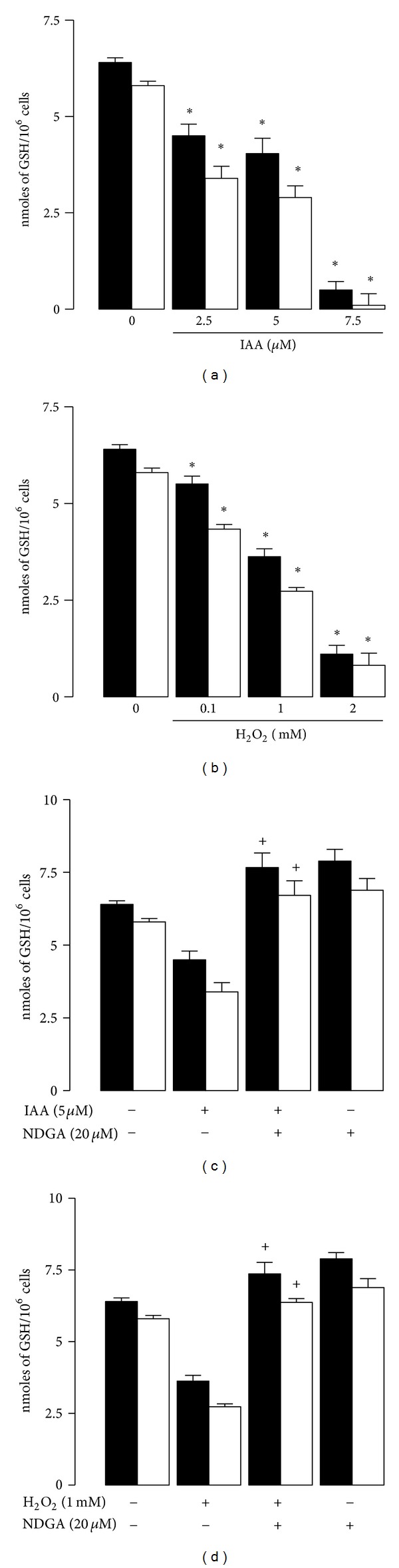
Effect of IAA or H_2_O_2_ on GSH + GSSG and GSH levels in THP-1 cells. Cells were treated 24 h with (a) IAA or (b) H_2_O_2_. After this time these compounds were withdrawn and fresh medium was added. GSSG and GSH levels were measured 24 h after the addition of toxic compounds. In addition the effect of NDGA on GSSG + GSH and GSH levels in (c) IAA or (d) H_2_O_2_ treated cells was also studied. Cells were treated with vehicle or 20 *μ*M NDGA by 12 h; after this the cells were exposed to 5 *μ*M IAA or 1 mM H_2_O_2_ for 24 h. GSSG and GSH levels were measured 24 h after the addition of toxic compounds. GSH levels were quantified by spectrophotometric/microplate reader assay. Data are expressed as mean ± SEM; *n* = 3.  *P < 0.05 versus control (without IAA or H_2_O_2_) and  ^+^ *P* < 0.05 versus the respective toxic compound.

 The effect of NDGA and toxic compounds (IAA or H_2_O_2_) on GSH levels was monitored by redox status in THP-1 cells (Figure 5). First, It was found that IAA or H_2_O_2_ induced reduction of [GSH] + [GSSG] and [GSH] concentrations in a concentration-dependent manner (Figures 5(a) and 5(b)) suggesting that both toxics caused an oxidative stress to the cells. In contrast, pretreatment of cells with 20 *μ*M NDGA (5 *μ*M IAA/NDGA or 1 mM H_2_O_2_/NDGA) abrogated the reduction of [GSH] + [GSSG] and [GSH] concentrations induced by oxidants. In addition, we also observed that NDGA alone caused slight increase of [GSH] + [GSSG] and [GSH] levels (Figures 5(c) and 5(d)).

### 3.4. CD33 Expression by IAA and H_2_O_2_ in Human Monocytes

**Figure 6:**
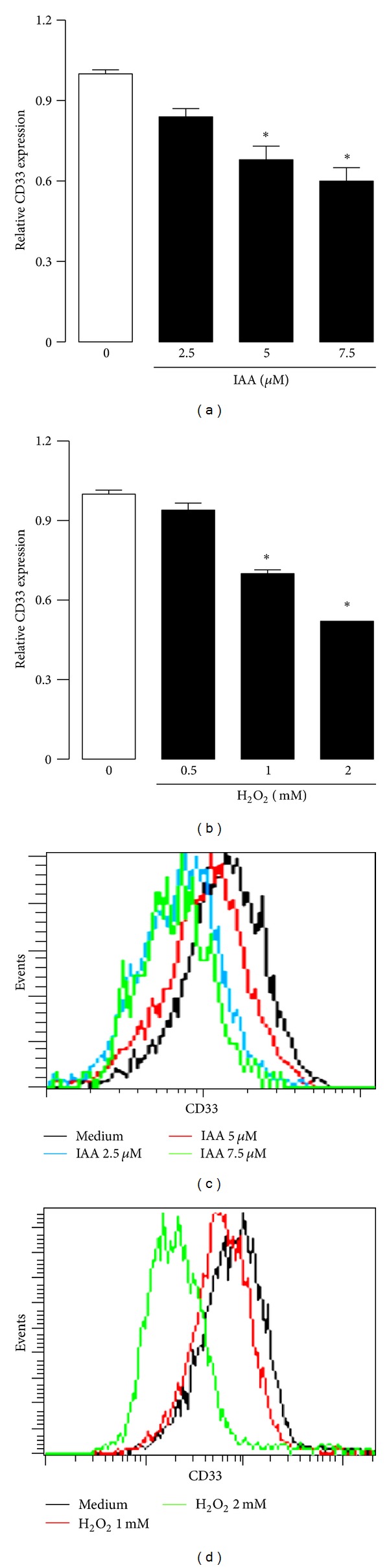
Effect of ((a), (c)) IAA or ((b), (d)) H_2_O_2_ on CD33 expression in MN. Cells were treated by 72 h with IAA or by 48 h with H_2_O_2_. Then, CD33 protein presence was determined by flow cytometry. The protein level was expressed as index with respect to control without treatment. Data are expressed as mean ± SEM; *n* = 5.  *P < 0.05 versus control (without IAA or H_2_O_2_).

We showed that treatment with either IAA or H_2_O_2_ significantly decreased CD33 expression levels in MNs. In IAA-treated cells (2.5, 5.0, and 7.5 *μ*M), CD33 expression levels decreased significantly compared to the control; this decrease in CD33 expression on the cell surface occurred in a concentration-dependent manner (Figures 6(a) and 6(c)). In MNs incubated with 1 and 2 mM H_2_O_2_, a significant reduction in CD33 expression was observed (Figures 6(b) and 6(d)). 

### 3.5. NDGA Effect on ROS Production and CD33 Expression in Human Monocytes

**Figure 7:**
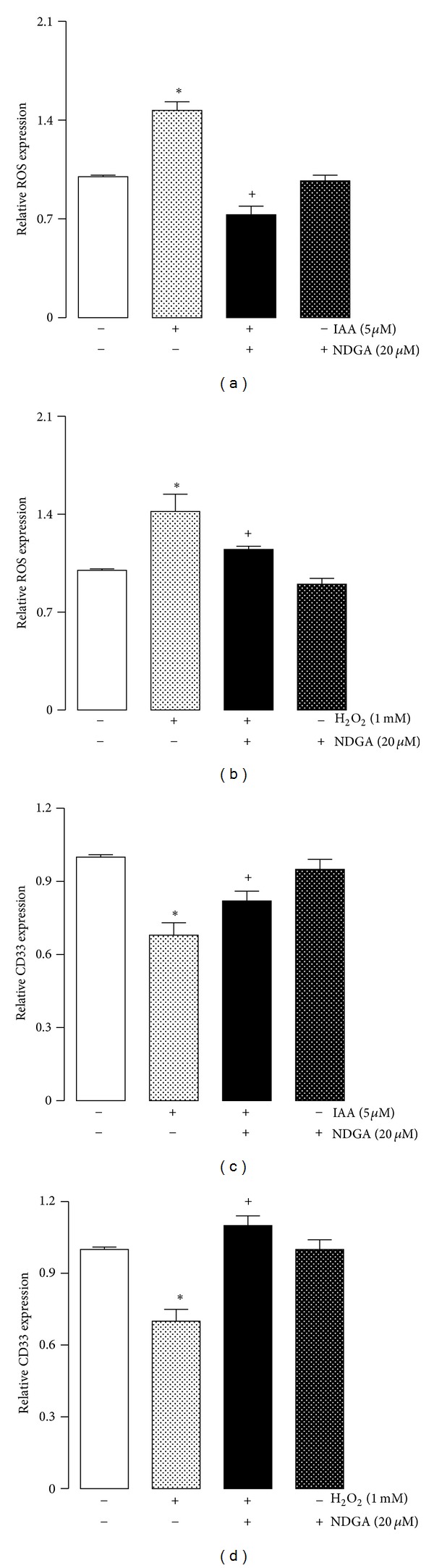
Effect of NDGA on ((a), (c)) IAA or ((b), (d)) H_2_O_2_ induced increase of ((a), (b)) ROS levels and ((c), (d)) decrease of CD33 expression in MN. Cells were pretreated with vehicle or 20 *μ*M NDGA for 12 h before the addition of 5 *μ*M IAA or 1 mM H_2_O_2_ and incubated for 72 and 48 h, respectively. ROS levels detection and CD33 expression were determined by flow cytometry and expressed as index compared to control without treatment. Data are expressed as mean ± SEM; *n* = 5.  ^+^ *P* < 0.05 versus control (without IAA or H_2_O_2_) and  *P < 0.05 versus control (without NDGA).

The protective effect of NDGA was evaluated with regard to H_2_O_2_- or IAA-induced ROS production and CD33 expression in MNs (Figure 7). NDGA was added to the culture prior to the oxidant exposure (5 *μ*M IAA and 1 mM H_2_O_2_), and it was maintained in the culture medium during the incubation. The ROS and CD33 expression levels were then measured by flow cytometry. 

NDGA prevented oxidative stress because the MNs cultured with NDGA/IAA (Figure 7(a)) or NDGA/H_2_O_2_ (Figure 7(b)) showed less ROS production. In addition, the decrease of CD33 expression was attenuated by NDGA in the cells exposed to IAA (Figure 7(c)) and H_2_O_2_ (Figure 7(d)). Notably, the cells exposed to NDGA had a similar CD33 protein expression level compared to the untreated control cells.

## 4. Discussion 

It is well established that NDGA has biological properties, such as anticarcinogenic [35–38], antidiabetic, antiviral, antioxidant, and anti-inflammatory activities in human cell cultures and animal models [3, 39]. Furthermore, NDGA has beneficial health properties, including the growth inhibition of human cancers *in vivo* [40, 41], the degradation of preformed Alzheimer's beta-amyloid fibrils *in vitro* [42], and the protection of cultured rat hippocampal neurons against the toxicity of the amyloid beta-peptide [43].

The purpose of this study was to evaluate the potential protective effects of NDGA on human MNs cultured under oxidative stress conditions. MNs are an essential host defense against microorganisms. MNs use mechanisms that consist of ingesting bacterial material through phagocytosis and killing infectious agents by producing ROS to protect the host [44]. Furthermore, ROS production performs other important physiological functions. For example, ROS participate in signal transduction and gene expression [45, 46]. MNs maintain intracellular redox homeostasis by balancing the production of ROS with their removal through cellular antioxidant defense systems.

However, excessive ROS production can be lethal for the MNs because ROS can attack biomolecules, which causes changes in the structure and function of these molecules. MNs are well known to play a crucial role in the development of ROS-induced pathologies because they can produce nonnegligible amounts of ROS. Because of the negative long-term side effects of ROS production by monocytes, modulating ROS generation and maintaining the redox state of the cell at the required physiological level are considered a main therapeutic target [25, 47]. NDGA has a protective effect due to its antioxidant capacity and has garnered increasing interest because it has been reported to contribute to the prevention or delay of oxidative stress-induced damage [4]. In this study, NDGA toxicity was first evaluated in human MNs. Our results demonstrated that, at concentrations of NDGA ranging from 5 to 25 *μ*M, NDGA is not cytotoxic to either THP-1 cells or human MNs over 120 h of treatment. This finding was comparable to animal cells, indicating that NDGA is nontoxic at low doses [4, 9, 10].

The potential protective effect of NDGA was then evaluated in two toxicity models using IAA and H_2_O_2_ to induce toxicity. Under these conditions, the oxidants caused cell death in a concentration-dependent manner in the THP-1 cells and human MNs. It was previously reported that these oxidants caused damage in primary cultures of rat neurons [9, 10].

IAA injury has been related to ROS production in primary cultures of rat neurons [8, 19]. It also has been demonstrated that in cultured hippocampal neurons, IAA reduces ATP levels and cell survival [18]. Our results suggest that IAA toxicity is related to the exacerbated ROS production and subsequent cell death of MNs (Figures 1(b) and 3(a)).

In contrast, H_2_O_2_ is a source of ^•^OH in the presence of transition metal ions. This oxygen metabolite reacts rapidly and broadly with all biomolecules. As expected, H_2_O_2_ caused increased ROS production and cell death [12]. In addition, it was recently described that H_2_O_2_ promotes the opening of the mitochondrial permeability transition pore (PTP), resulting in membrane depolarization, uncoupling of oxidative phosphorylation, and potential cell death in porcine LLC-PK1 cells [11].

In addition, we demonstrated that NDGA protected MNs and THP-1 cells against H_2_O_2_. We observed that treatment with NDGA prior to the toxic challenges induced by IAA and H_2_O_2_ significantly diminished the toxicities of these compounds. In this context, it has been shown that the protective effect of NDGA is predominantly due to its antioxidant capacity. In fact, the direct ROS scavenging capacity and induction of antioxidant enzymes via the Nrf2 pathway may be involved in the mechanism by which NDGA exerts its protective effect [4, 9–11]. Recently, it was described that NDGA can prevent the mitochondrial damage that is induced by oxidative stress in renal epithelial LLC-PK1 cell cultures [11] and in an animal model of renal damage [48]. 

We also explored the effect of NDGA on the oxidant stress-induced downregulation of CD33 expression in human MNs. Our results indicated that NDGA is a potent antioxidant that can prevent low levels of oxidative stress and can also prevent the decrease in CD33 expression in cells treated with IAA and H_2_O_2_. 

The mechanisms involved in the expression of CD33 have not been fully elucidated. But have been described two mechanisms of repressive control [49, 50]. First, CD33 activity is decreased by SOCS3, which is a member of the suppressor of cytokine signaling (SOCS) protein family. The binding of SOCS3 to the phosphorylated immunoreceptor tyrosine-based inhibitory motifs (ITIM) of CD33 induces the proteosomal degradation of both molecules and the reduction of CD33 expression on surface monocytes and blockades the increased secretion of IL-1*β*, IL-8, and TNF-*α* [49, 51]. The second mechanism described in myeloma cells was induced by IL-6. This cytokine upregulates the expression of helix-loop-helix leucine zipper transcription factor (MYC) via transcriptional regulator of the immune response 3 (STAT3) phosphorylation. MYC binds directly to the promoter region of the CCAAT enhancer binding protein *α* (C/EBPA gene), which downregulates C/EBPA and thus CD33 gene expression is decreased [50]. Finally, Gonzalez et al. (2012) showed that hyperglycemia diminished both mRNA and CD33 expression in surface cell. But, when the human monocytes were treated with *α*-tocopherol, this negative modulation was prevented. In fact, it is well known that SOCS3 is modulated by oxidative stress in response to hyperglycemia [52, 53] and the TNF-*α* production is induced by H_2_O_2_ via oxidative stress-related signal pathways [54]. Because our previous study found that the antioxidant *α*-tocopherol prevents TNF-*α* production and CD33 downregulation, therefore it is possible that ROS induction could be participating in these processes.

Furthermore, MYC is induced by oxidative stress generated by sodium arsenate in the cell line MCG-7 [55]. Even C/EBPA is susceptible to negative regulation by oxidative stress induced by H_2_O_2_ in 3T3-L preadipocyte cells [56] and by ethanol in HepG2 cells [57]. In addition, we observed a significant decrease of CD33 protein in surface cells in oxidative condition induced by IAA and H_2_O_2_. This result supports the idea that oxidative stress could alter the transcription of CD33 by modulating the transcription factors such as STAT, MYC, and C/EBPA.

Preliminary studies in our research group demonstrated that oxidative stress induced by hyperglycemia decreases CD33 expression in human monocytes, but the pretreatment with the antioxidant *α*-tocopherol prevents ROS production and alteration in the CD33 expression. NDGA and *α*-tocopherol prevent ROS generation. *α*-Tocopherol inhibits superoxide anion production by impairment of the NADPH oxidase assembly and inhibits p47phox translocation to the membrane [58]. Moreover, NDGA is a selective inhibitor of 12-lipoxygenase (12-LOX), which produces ROS during arachidonic acid metabolism. The mechanism by which ROS induced CD33 downregulation could be through the induction of inflammation, because IAA or H_2_O_2_ induces the inflammatory cytokine TNF-*α*, and NDGA is a powerful antioxidant compound that affects a wide variety of cellular processes including TNF-*α*. In this work, NDGA prevented the decrease in the expression of CD33 secondary to oxidative stress induced by H_2_O_2_ or IAA. These data suggest that alterations in CD33 secondary to oxidative conditions may be counteracted by exogenous antioxidants of different structure such as *α*-tocopherol or NDGA. In addition, low concentration of NDGA may contribute to decreased oxidative stress by either scavenging ROS [4] and/or by the induction of Nrf2-dependent antioxidant enzymes [9, 11] and avoiding inflammation as inhibitor or COX-2 and LOX [39].

In conclusion, this study presents novel findings supporting the ameliorative effect of NDGA on the oxidant condition of human MNs. NDGA could prevent cell death under severe oxidative stress conditions. There was a slight increase of ROS production induced by H_2_O_2_ and IAA and a significant decrease of CD33 expression on MN surfaces. However, NDGA prevented these negative effects. These results suggest that changes in the redox state induced by hyperglycemia, IAA, or H_2_O_2_ generate an important signal that causes CD33 modulation in a negative manner, and this state might contribute to MN activation.
